# Reliability of a non-invasive method to calculate buffer capacity after exhaustive cycling exercise of 20 s to 12 min: a pilot study

**DOI:** 10.3389/fspor.2025.1546117

**Published:** 2025-03-12

**Authors:** Sebastian Gehlert, Asatur Khurshudyan, Sebastian Weber, Jochem Widdershoven, Reinout Van Schuylenbergh

**Affiliations:** ^1^ Institut für Sportwissenschaft, University of Hildesheim, Hildesheim, Lower Saxony, Germany; ^2^ INSCYD GmbH, Salenstein, Switzerland; ^3^ Faculty of Kinesiology and Rehabilitation Sciences, KU Leuven, Leuven, Belgium

**Keywords:** buffer capacity, exercise induced acidosis, lactate, reliability, cycling

## Abstract

Traditionally, buffer capacity (*β*) is measured on muscle biopsies by measuring changes in muscle pH in relation to exposure of standardized quantities of hydrochloric acid. This is an invasive approach requiring specific equipment and trained personnel which limits its usability in a normal training context. Assessing *β* using capillary blood lactate concentration (BLC) and pH values has been proposed as a more practical and cost-effective approach. The reliability of the input BLC and pH data on the calculations of *β* after maximal sprint and endurance exercise has not yet been investigated and was major aim of our study. Eleven subjects performed six maximal performance tests ranging from 20 s to 12 min duration over a 2-week period. All subjects were familiarized with the test conditions. For each performance test, pre and posttest BLC and pH was measured and used to calculate *β* using the Henderson-Hasselbach equation. As BLC_pre_ and pH_pre_ values showed poor reliability, *β* calculations were repeated using constants for BLC_pre_ (1.23 mmol·L^−1^) and pH_pre_ (7.426) chosen from the average values in the experimental data. Test-retest reliability for BLC_pre_ (ICC: 0.12, 95% CI −0.49–0.65, n.s.) and pH_pre_ (ICC: 0.40, 95% CI −0.22–0.79, n.s.) was poor, whereas BLC_post_ (ICC: 0.95, 95% CI 0.82–0.99, *p* < 0.05) and pH_post_ (ICC: 0.89, 95% CI 0.65–0.97, *p* < 0.05) displayed good to excellent reliability. Good reliability was observed for *β* calculated from the Henderson-Hasselbalch equation utilizing BLC_post_ and pH_post_ only (ICC: 0.86, 95% CI 0.55–0.96, *p* < 0.05). The validity of this method in comparison with gold-standard methods needs further scientific investigation.

## 1 Introduction

Intense muscular activity involving a high energy contribution from non-oxidative pathways, is associated with large ionic changes in the muscle, including the accumulation of hydrogen ions (H^+^). While the role H^+^ has in causing muscle fatigue has been questioned (1–3) and is still under investigation, the accumulation of H^+^ has been shown to contribute to a reduction in exercise performance by affecting muscle fatigue (4, 5) and the perception of effort (central fatigue) (6). Proton accumulation depends on both the production and removal of H^+^. The intra- and extracellular buffer systems act to reduce the buildup of free H^+^ and reduces the fluctuations in cellular pH during high-intensity (non-steady-state) exercise (7). Muscle buffer capacity (*β*_m_) has been reported to be higher in highly trained team-sport athletes, sprinters and rowers than in marathon runners or untrained subjects (8, 9). This suggests that athletes involved in sports with a greater anaerobic demand may have a greater *β*_m_. However, the results of cross-sectional studies do not allow us to conclude whether this greater *β*_m_ is due to event selection, or the type of training undertaken. Buffer capacity can be improved by specific training. High-intensity training with bouts of 120%–140% of the anaerobic threshold has been shown to improve *β*_m_ with 17%–25% (10, 11) whereas moderate intensity training (11) or high-repetition resistance training (12) did not improve *β*_m_. Pretraining *β*_m_ negatively correlated with changes in *β*_m_ following high-intensity training (13). Increasing *β*_m_ by consuming sodium bicarbonate has been shown to be effective in improving buffer capacity and maximal exercise performance of 30 s to 12 min duration, typically associated with high accumulation of H^+^ (14–18). The ergogenic effects of sodium bicarbonate supplements seems to be more pronounced in individuals with lower *β*_m_ or training status (19). Therefore, knowing the individual's *β*_m_ provide essential information to make informed decisions on training or supplement interventions.

Traditionally, *β*_m_ is measured on muscle biopsies by measuring changes in muscle pH in relation to the exposure of standardized quantities of hydrochloric acid (HCl). From the fitted titration trend line, the subject's *β*_m_ is calculated (20). Alternatively, *β*_m_ can also be assessed based on the ratio of the pre-post exercise lactate concentration (Δlactate) over the pre-post exercise pH (ΔpH) in muscle tissue (8, 21). Skeletal muscle ^31^P magnetic resonance spectroscopy (^31^P MRS) can be utilized as a non-invasive approach to investigate muscle metabolism in resting and exercising subjects (22, 23). Unfortunately, all these measuring techniques of *β*_m_ are expensive, invasive (muscle biopsies), time consuming and require laboratory equipment and trained personnel to perform the measurements and data analysis. Consequently, these methods are hardly applicable in the context of physical training and performance monitoring of athletes. Sahlin et al. demonstrated that blood lactate and blood pH mimics the behavior of lactate and pH in the exercising muscle tissue (21). This can be explained by the co-transport of lactate and H^+^ from the intracellular to the extracellular space by the lactate-proton transporters, monocarboxylate transporter 1 (MCT1) and 4 (MCT4) (24–26). The lactate/H^+^ co-transport seems to be the most important system to remove H^+^ from the intracellular to the extracellular space during high-intensity exercise (24, 25). The intracellular accumulation of H^+^ depends on the extracellular H^+^ concentration. H^+^ efflux out of the muscle cell has been reported to be inhibited by extracellular acidosis (27) and enhanced by a greater extracellular buffer concentration (28). Therefore, lactate and H^+^ accumulation in the blood might be useful markers for lactate and H^+^ accumulation in the exercising muscle and valid input data to calculate *β* (29). Whereas traditional measuring techniques of *β* are expensive, invasive, time consuming, and therefore, are difficult to implement during physiological monitoring of athletes, assessing *β* from pH and lactate in capillary blood samples might overcome such issues.

To obtain reliable calculations of *β*, the reliability of input data must be ensured. Sources of variability are found in the analyzer used and in biological variability. The reliability and validity of lactate analyzers is extensively investigated (30–32). The within-sample standard deviations for handheld lactate analyzers are in general <0.5 mmol·l^−1^ with greater imprecision (>1 mmol·l^−1^) for lactate concentrations >15 mmol·l^−1^ (30, 31, 33) as can be observed after short maximal exercise. The biological variability in the pre-exercise lactate response is ∼30% (34, 35). How these sources of variability affect the calculation of *β* is not yet investigated.

The purpose of this study is to investigate the test-retest reliability of the variables for calculating *β* (i.e., pre- and posttest blood lactate concentration and pH). We hypothesize that these input variables display robust reliability allowing for further calculation of *β*. Secondly, we investigate the consistency of the calculation of *β* under varying test conditions. We hypothesize that the calculation of *β* is independent of the test duration in maximal, non-steady state efforts.

## 2 Materials and methods

### 2.1 Subjects

Eleven endurance trained male subjects (mean ± SD: age 32.2 ± 15.2 years, mass 70.9 ± 6.7 kg, height 180.4 ± 6.6 cm, body fat 14 ± 5.6%, $\dot{V}O_{2\max}$ 4.18 ± 0.48 L·min^−1^, $vLa_{\max}$ 0.46 ± 0.08 mmol·L^−1^·s^−1^) gave their informed written consent to take part in the study. All subjects were familiar with laboratory exercise testing on the cycle ergometer. The study protocol was approved by the ethics committee of the Hildesheim University, Germany.

### 2.2 Study protocol

**Figure 1:**
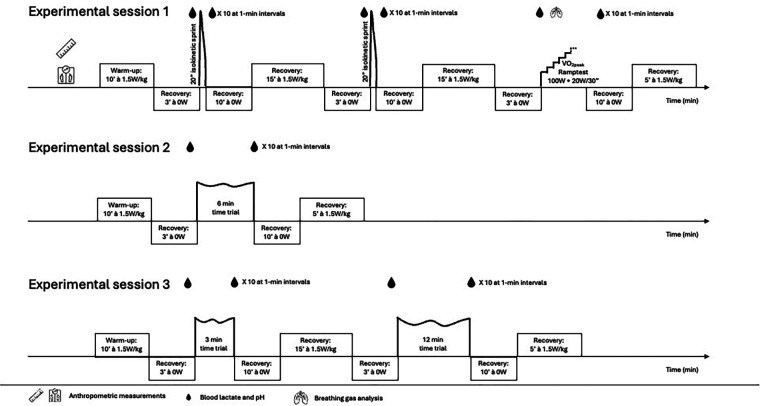
Overview of the experimental tests.

The subjects performed three experimental sessions over a two-week period and completed six maximal performance tests of 20 s to 12 min duration (Figure 1). These test durations were selected because they are associated with large accumulation of hydrogen ions (H^+^) in the muscle and blood and are affected by alterations in buffer capacity (16–18). The day before the experiments the subjects were allowed to perform maximum of 1 h of low-intensity training. Furthermore, the subjects were encouraged to consume ∼3 h before the exercise test a standardized meal (∼2,700 kJ; 71% carbohydrate, 15% fat, 14% protein). To avoid fluctuations of initial muscle glycogen content, they were asked to adopt an identical training and food regimen for all subsequent experimental exercise sessions (36). To account for diurnal variation, subjects reported to the experimental sessions at the same time of the day. All cycling tests were performed on the subject's personal racing bike mounted on a calibrated cycle ergometer (Cyclus 2, Leibzig, Germany). The Cyclus 2 is an electromagnetically braked ergometer and measures power output with an accuracy error of 2% according to the manufacturer. The gear ratio was fixed at either 53 × 12 or 52 × 12, depending on the front chainring of the subject's bicycle. The cycling tests were performed in an air-conditioned exercise laboratory (19°C, 60% relative humidity) and supervised by trained personnel. The subjects were verbally encouraged to perform all tests with maximal effort. During all tests, air ventilation was provided to assist body cooling (37).

#### 2.2.1 Experimental session 1

At the first visit to the laboratory, the subjects body length, body mass and body composition (Seca, BCA 525, Hamburg Germany) were measured. After these measurements, subjects performed a 10 min warm-up at a self-selected cadence at 1.5 W·kg^−1^. This warm-up phase was followed by 3 min of passive recovery to reduce the aerobic energy contribution in the subsequent sprint. A capillary blood sample was taken from a hyperaemic earlobe (Finalgon®, Frankfurt, Germany) to determine pre-test blood lactate concentration (BLC_pre_) (EKF, Biosen C-line, Barleben, Germany) and blood pH (pH_pre_) (Radiometer ABL 90 Flex, Copenhagen, Denmark).

The first test consisted of a 20 s isokinetic sprint at 120 rev·min^−1^. During the sprint subjects had to maintain a seated position. The initial load of the ergometer was set at three times the fat free mass of the subjects (in Newton) and was activated at 60 rev·min^−1^ (SPR1). After the sprint the subjects rested 10 min seated on the saddle, without pedaling. During this period of passive rest, post-test capillary blood samples were taken at 1-min intervals to capture the highest lactate (BLC_post_) and lowest pH (pH_post_) values. The maximal lactate accumulation rate $\left(vLa_{\max}\right)$ was calculated using the formula below (38, 39):

$$vLa_{\max}=\;\frac{\mathrm{BL}C_{\mathrm{post}}-\mathrm{BL}C_{\mathrm{pre}}}{20\;s-\;t_{\mathrm{alac}}}$$

*T*_alac_ = time (s) from time 0 to 3% drop in peak power (39, 40).

Thereafter, the subjects cycled for minimum 15 min at 1.5 W·kg^−1^ to promote recovery between tests. The level of recovery was controlled by measuring BLC. Subjects with BLC exceeding 2 mmol·L^−1^ extended active recovery with 3 min. To assess test-retest reliability, the isokinetic test was repeated with identical ergometer settings, post-exercise recovery procedures and BLC and pH measurements (SPR2). After these 2 sprint-tests a ramp protocol was performed to assess $\dot{V}O_{2\max}$. The test started at 100 W. Workload increased every 30 s with 20 W until volitional exhaustion. The subjects were allowed to use their preferred cadence (97 ± 7 rev·m^−1^). Breathing gases were analyzed (Omnical Sport, Maastricht Instruments, The Netherlands) and heart rate recorded (Polar, Kempele, Finland). A maximal effort was established when 2 or more of the following criteria were met (41):

• Plateau in $\dot{V}O_{2}$ (<120 ml·min^−1^) despite an increase in workload
• Maximal heart rate within 10 beats of age predicted maximal heart rate (220-age)
• RER > 1.1
• BLC > 8.0 mmol·L^−1^

#### 2.2.2 Experimental session 2

On the second laboratory visit, subjects performed a 10 min warm-up at a self-selected cadence at 1.5 W·kg^−1^. At the end of the warm-up, a capillary blood sample was taken from a hyperaemic earlobe to determine BLC_pre_ and pH_pre_. Thereafter, a 6 min maximal time trial (TT_6_) was performed. The subjects were instructed to achieve the highest possible average power output for the given test duration. The subjects could adjust their workload intensity (5 W steps) at any time. The subject performed the test in the seated position. They used the same pedaling frequency as from the ramp test (experimental session 1). After the time trial, the subjects rested 10 min seated on the saddle, without pedaling. During this period of passive rest, post-test capillary blood samples were taken at 1-min intervals for BLC_post_ and pH_post_ determination.

#### 2.2.3 Experimental session 3

On the third laboratory visit, subjects performed a 10 min warm-up at a self-selected cadence at 1.5 W·kg^−1^. At the end of the warm-up, a capillary blood sample was taken from a hyperaemic earlobe to determine BLC_pre_ and pH_pre_. The subjects completed two maximal time trials of 3 min (TT_3_) and 12 min (TT_12_) duration, interspersed with ∼25 min recovery. The subjects were instructed to achieve the highest possible average power output for the given test duration. The subjects could adjust their workload intensity (5 W steps) at any time. The subject performed the test in the seated position. They used the same pedaling frequency as from the ramp test (experimental session 1). After each time trial, the subjects rested 10 min seated on the saddle, without pedaling. During this period of passive rest, post-test capillary blood samples were taken at 1-min intervals for BLC_post_ and pH_post_ determination. Thereafter, the subjects cycled for minimum 15 min at 1.5 W·kg^−1^ to promote recovery between tests. The level of recovery was controlled by measuring BLC. Subjects with BLC exceeding 2 mmol·L^−1^ extended active recovery with 3 min.

### 2.3 Calculation of buffer capacity

The buffer capacity (*β*) was calculated according to Equations 1, 2, using the Henderson-Hasselbalch equation (42). The Henderson-Hasselbalch equation is a mathematical formula that describes the relationship between the pH of a solution, the dissociation constant of an acid (pKa), and the ratio of the conjugate base to the undissociated acid. It is frequently used in biochemistry to calculate the pH of buffer systems. The dissociation constant (pK_a_) for lactate was set at 3.87. The transformation of the Henderson-Hasselbalch equation into the equation applied in this study is presented in the Supplementary Materials.

(1) $$\mathrm{Method}\;1:\;\beta 1=\;\frac{\left(\frac{\mathrm{BL}C_{\mathrm{post}}}{10^{\left(pH_{\mathrm{post}}-3.87\right)}}\;-\;\frac{\mathrm{BL}C_{\mathrm{pre}}}{10^{\left(pH_{\mathrm{pre}}-3.87\right)}}\right)}{pH_{\mathrm{post}}-\;pH_{\mathrm{pre}}}$$

Pre-examination of the BLC_pre_ and pH_pre_ data revealed low test-retest reliability (Table 1). Therefore, we calculated *β* with the measured pretest values as well as with a constant BLC_pre_ and pH_pre_. For this purpose, we used the average BLC_pre_ (1.23 mmol·L^−1^) and pH_pre_ values (7.426) from all tests, to obtain representative constants (Equation 2).

(2) $$\mathrm{Method}\;2:\;\beta 2=\;\frac{\left(\frac{\mathrm{BL}C_{\mathrm{post}}}{10^{\left(pH_{\mathrm{post}}-3.87\right)}}\;-\;\frac{1.23}{10^{\left(7.426-3.87\right)}}\right)}{pH_{\mathrm{post}}-\;7.426}$$

**Table 1:** Intraclass correlation coefficients (ICC) (95% CI): Pre-exercise blood lactate concentration (BLC_pre_), pre-exercise pH (pH_pre_), post-exercise BLC (BLC_post_) and pH (pH_post_). SPR1, first isokinetic sprint; SPR2, second isokinetic sprint; (details see methods section).

SPR1—SPR2	Delta	ICC	Effect	95% CI
BLC_pre_	0.56 ± 0.57 mmol·l^−1^	0.12	Poor	−0.49 to 0.65
BLC_post_	−0.4 ± 0.57 mmol·l^−1^	0.95*	Excellent	0.82–0.99
pH_pre_	0.016 ± 0.024	0.40	Poor	−0.22 to 0.79
pH_post_	0.005 ± 0.013	0.89*	Good	0.65–0.97

* *p* < 0.05.

### 2.4 Statistical analysis

Raw data was processed using Microsoft Excel (version 16.92, Redmond, USA). Statistical analyses (Mean, standard deviations, repeated measures ANOVA) were performed using GRAPHPAD PRISM (version 10 GraphPad Prism Software, Boston, USA). The Shapiro Wilk test was used to evaluate the normality of the data. Descriptive statistics were calculated for all variables and presented as mean ± standard deviation (SD).

The reliability of the *β* calculations depends on the test-retest reliability of the input data. Therefore, the reliability of pre and post BLC and pH values for the sprint tests was quantified using intraclass correlation coefficients (ICC) (JASP statistical software, version 0.19 for Apple Silicon, The Netherlands). The reliability of the *β* calculations for the sprint tests was assessed using ICC. ICC values less than 0.5, between 0.5 and 0.75, between 0.75 and 0.9 and greater than 0.9 are indicative of poor, moderate, good and excellent reliability, respectively (43).

The consistency of the pre and post BLC and pH values and *β*1 and *β*2 under varying test conditions was evaluated with a repeated measures ANOVA and Pearson correlation coefficients. Bland–Altman plot analysis (expressed as the mean ± 1.96 SD) was used to quantify the bias and the range of agreement between SPR1 and SPR2 for BLC_pre_, BLC_post_, pH_pre_, pH_post_, *β*1 and *β*2 with 95% limits of agreement.

A probability level of *p* < 0.05 was set as the criterion for acceptance of statistical significance.

## 3 Results

### 3.1 Reliability of input data and buffer capacity

**Figure 2:**
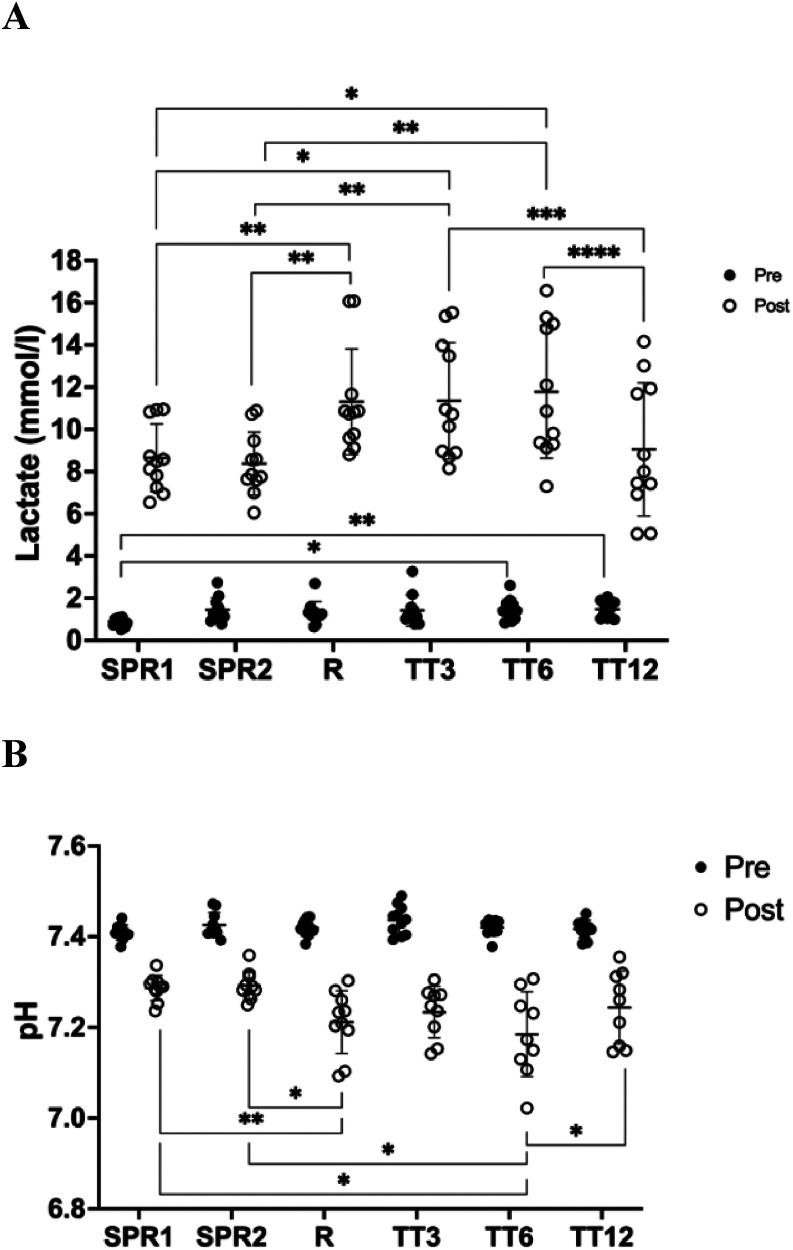
Blood lactate concentration (BLC) **(A)** and pH **(B)** (mean ± SD). SPR1, first isokinetic sprint; SPR2, second isokinetic sprint; Ramp, maximal ramp test protocol; TT_3_, 3 min maximal time trial; TT_6_, 6 min maximal time trial; TT_12_, 12 min maximal time trial (details see methods section). Repeated measures ANOVA: *p < 0.05, **p < 0.01, ***p < 0.001.

Mean values (±SD) for the measured BLC and pH data are shown in Figure 2. BLC_pre_ for the first sprint test was lower compared to the other performance tests (Δ ∼ 0.4 mmol·L^−1^). PH_pre_ values were similar between the different test conditions (∼7.425). BLC_post_ were similar in the sprint tests and TT_12_ (∼8.6 mmol·L^−1^) and lower than for the other endurance tests (∼11.5 mmol·L^−1^). An inverse pattern was observed for pH_post_ values, with pH values for the ramp test and TT_3_ and TT_6_ being significantly lower (∼7.2) compared to the pH_post_ after the sprint tests and TT_12_ (∼7.28).

Test-retest reliability for BLC_pre_ (ICC 0.12, 95% CI −0.49–0.65, n.s.) and pH_pre_ (ICC 0.40; 95% CI −0.22–0.79, n.s.) was poor. Test-retest reliability for BLC_post_ (ICC 0.95, 95% CI 0.82–0.99, *p* < 0.05) and pH_post_ were good to excellent (ICC0.89, 95% CI 0.65–0.97, *p* < 0.05) (Table 1).

Test-retest reliability (ICC) for *β*1 was poor (ICC 0.26, 95% CI −0.38–0.73) n.s.). Good reliability was observed for *β*2 (ICC 0.86, 95% CI 0.55–0.96, *p* < 0.05) (Table 2).

**Table 2:** Intraclass correlation coefficients (ICC) of calculated buffer capacity (*β*1 and *β*2). SPR1, first isokinetic sprint; SPR2, second isokinetic sprint; (details see methods section).

SPR1—SPR 2	Delta	ICC	Effect	95% CI
*β*1	−44 ± 46 μmol·l^−1^·pH unit^−1^	0.26	Poor	−0.38 to 0.73
*β*2	−5 ± 18 μmol·l^−1^·pH unit^−1^	0.86*	Good	0.55–0.96

* *p* < 0.05.

### 3.2 Consistency of buffer capacity under varying test conditions

**Figure 3:**
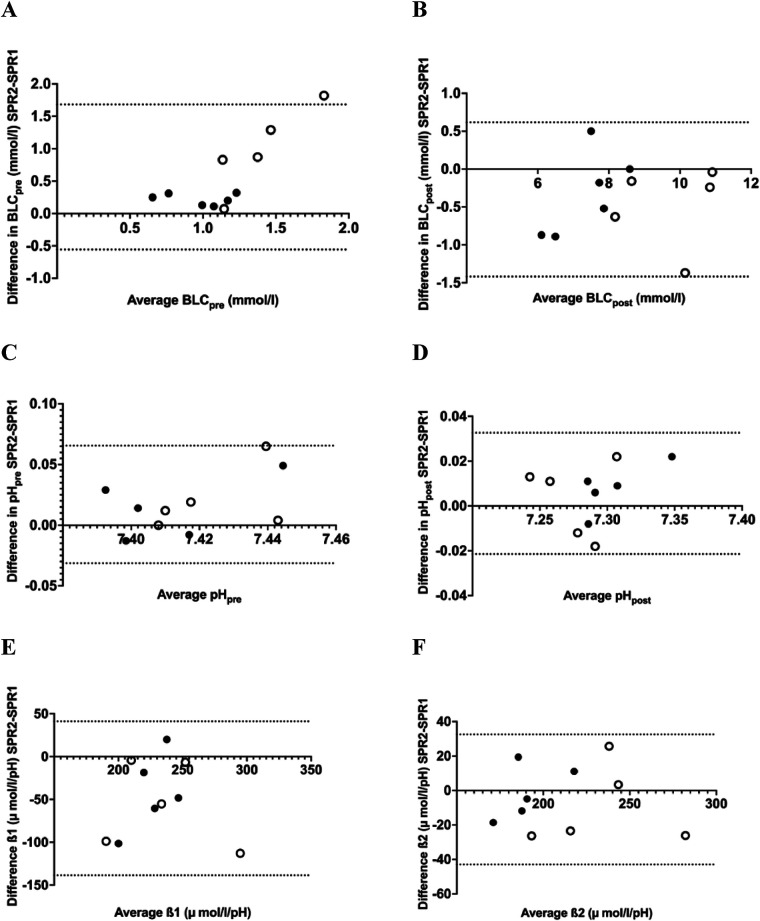
Bland-Altman plots comparing SPR1 and SPR2 for pre-exercise blood lactate concentration (BLC) **(A)**, post-exercise BLC **(B)**, pre-exercise pH **(C)** post-exercise pH **(D)** buffer capacity (*β*1) **(E)** and *β*2 **(F)** open symbols represent subjects with *ν*La_max_ ≥ 0.5 mmol·L^−1^·s^−1^.

*β*1 ranged between 210 and 255 μmol·l^−1^·pH^−1^ for the sprint tests and between 207 and 245 μmol·l^−1^·pH^−1^ for the endurance tests, without significant differences between the tests (Figure 3A). *β*2 ranged from 209 to 245 μmol·l^−1^·pH unit^−1^. *β*2 was significantly higher in TT_6_ compared to TT_12_ (Δ 31 μmol·l^−1^·pH unit^−1^) and similar in all other conditions (Figure 3B).

Buffer capacity (*β*1 and *β*2) derived from the time trial tests is strongly correlated (*r* = 0.60–0.86, *p* < 0.05) (Supplementary Figures S1A,B). No significant correlations were observed between the buffer capacity calculated from the time trial tests in comparison with the buffer capacity calculated from the sprint test (*r* = 0.18–0.51, n.s.).

## 4 Discussion

The present study was designed to evaluate the test-retest reliability of pre- and posttest BLC and pH to calculate extracellular buffer capacity (*β*) during maximal cycling exercise. Secondly, we investigate the consistency of the calculation of *β* under varying test conditions. The main findings of the present investigation are:

(i) We observed poor reliability for the pretest BLC and pH data.
(ii) We observed good to excellent reliability for the posttest BLC and pH data.
(iii) We observed good reliability for *β* utilizing BLC_post_ and pH_post_ only (*β*2)
(iv) The calculated *β* was similar between the test conditions, however *β* derived from the sprint tests or time trial tests cannot be used interchangeably.

### 4.1 Reliability of input data

**Figure 4:**
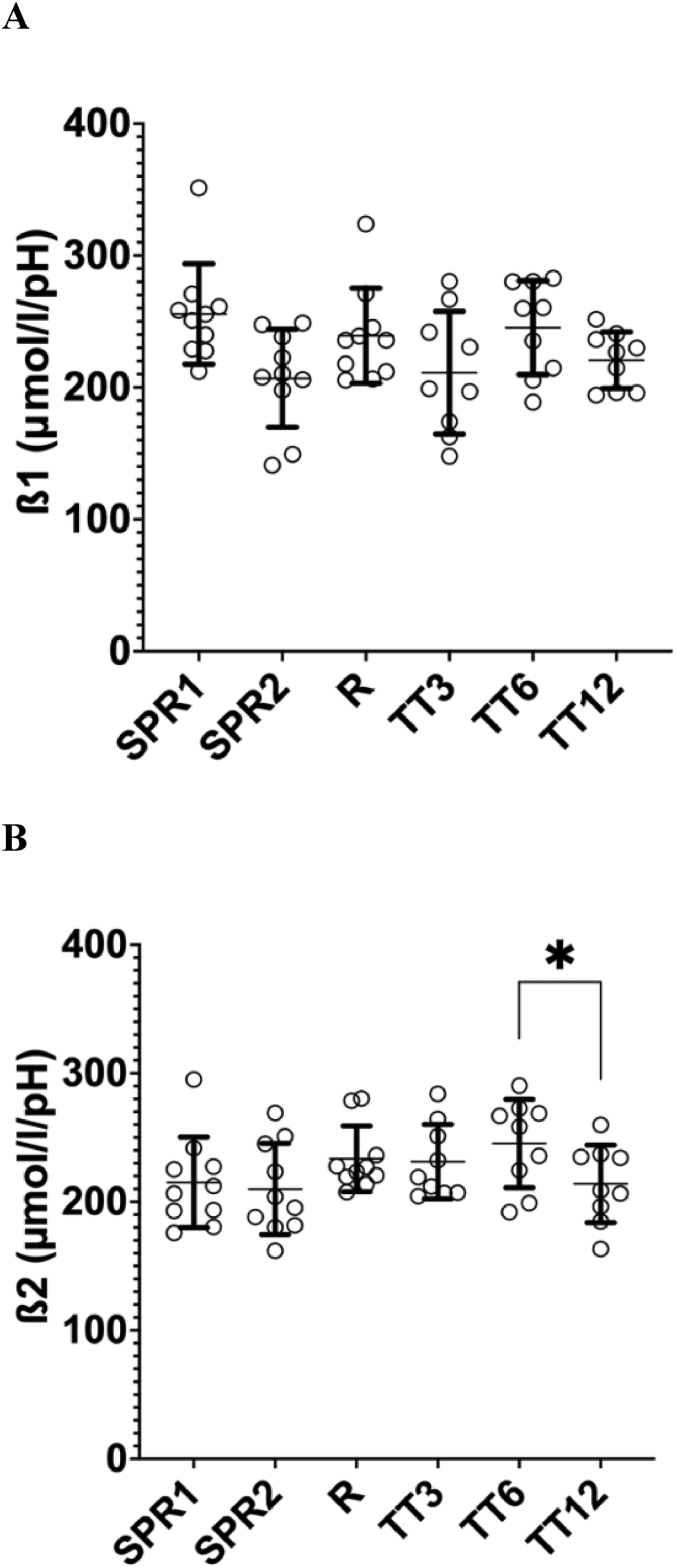
Buffer capacity calculated with method 1 (*β*1) **(A)** and method 2 (*β*2) **(B)** (mean ± SD). SPR1, first isokinetic sprint; SPR2, second isokinetic sprint; Ramp, maximal ramp test protocol; TT_3_, 3 min maximal time trial; TT_6_, 6 min maximal time trial; TT_12_, 12 min maximal time trial (details see methods section). Repeated measures ANOVA: *p < 0.05.

The BLC_pre_ ranged between 0.5–2.7 mmol·l^−1^ and BLC_post_ ranged between 5.7–10.9 mmol·l^−1^ which was comparable to earlier observations in maximal sprint tests of 10–30 s duration in physically active individuals (44–46). Closer examination of our data revealed that in 4 of the 5 subjects with a *ν*La_max_ ≥ 0.5 mmol·L^−1^·s^−1^, had elevated BLC_pre_ in the second sprint (0.8–1.8 mmol·L^−1^) whereas this was not observed in the subjects with *ν*La_max_ < 0.5 mmol·L^−1^·s^−1^ (Figure 4A). Apparently in these subjects 10 min of passive followed by ∼20 min of active recovery was not sufficient to restore the BLC to pre-test levels. This result exceed the biological variability of ∼30% for pre-exercise BLC that have been reported in the literature (34, 47) and may have contributed to the reduced reliability in the pre-test BLC.

The pH_pre_ values ranged between 7.378 and 7.472, in line with literature data (9, 15, 47). The pH_pre_ was not significantly different between trials, however displayed poor reliability. This is not entirely surprising given the small interindividual variation in baseline pH (9, 47, 48) which might affect the correlation (49). Average pH_post_ values ranged between 7.185–7.292, with the lowest value observed in TT_6_. These data correspond well with earlier observations after supra-maximal exercise (9, 50, 51). Our data showed good reliability for pH_post_.

### 4.2 Test consistency for buffer capacity determination

The buffer capacity (*β*) was calculated from pre- and posttest BLC and pH data using the Henderson-Hasselbalch equation (42). The Henderson-Hasselbalch equation is a mathematical formula that describes the relationship between the pH of a solution, the dissociation constant of an acid (pKa), and the ratio of the conjugate base to the undissociated acid. The average buffer capacity calculated from this method (*β*1) ranged between 207 and 255 μmol·l^−1^·pH^−1^, without significant differences between the tests. As the resting values for BLC and pH had poor reliability, we recalculated the buffer capacity replacing this data with the average BLC_pre_ and pH_pre_ values (method *β*2). The average buffer capacity calculated from this method (*β*2) yield similar values compared to *β*1, except for the first sprint test (Δ 40 μmol·l^−1^·pH^−1^). Normative data on muscle buffer capacity (in µmol H^+^·g muscle dry-weight^−1^·pH^−1^) is abundantly available in the scientific literature in non-exercising subjects (8, 9), sprint-, teamsport- (9) and endurance athletes (9, 10, 52). Muscle buffer capacity is typically higher in athletes compared to non-athletes, and sprint athletes compared to endurance athletes (9). To the best of our knowledge, reference data for extracellular buffer capacity is not yet documented in the scientific literature. Therefore, the data in our study cannot be compared to literature data.

### 4.3 Strengths and limitations

Our study provided quantitative data on the reliability of BLC and pH measurement pre and post exercise for the calculation of extracellular buffer capacity. We consider our methodological approach to ensure high data quality as a strength of this study. We tested trained cyclists familiar with maximal exercise testing in a laboratory to prevent learning or training effects. The measurements were performed by trained personal using bench-top lactate and pH analyzers to minimize sources of measurement error. It has repeatedly been shown that handheld lactate analyzers lack the accuracy of bench-top machines (30–32). Most (30, 32), but not all handheld lactate analyzers (31, 32) are undersensitive at higher lactate concentrations (above ∼8 mmol·L^−1^) and this could jeopardize the calculation of buffer capacity. For example, using our mathematical method, a difference of 1 mmol·l^−1^ in post exercise BLC would result in ∼12% difference in *β* and almost double the typical error of the titration technique on muscle biopsies (53). This means that the proposed mathematical method is sensitive to the quality of the input data and therefore lab accurate equipment is recommended to ensure good data quality. The use of bench-top analyzers can also be seen as a limitation of our experimental methodology as such expensive equipment limit the useability in the sports training context.

Some limitations of this study should be acknowledged. In 35% of our subjects 10 min of passive followed by ∼20 min of active recovery was not sufficient to restore the BLC to pre-test levels. It has repeatedly shown that active recovery is effective to accelerate BLC recovery to pre-test levels (54–56). For instance Menzies et al. demonstrated that 20 min at 80%–100% of the lactate threshold intensity (LT1) was sufficient to restore BLC to pre-test levels, but longer recovery times were needed at lower exercise intensities (56). In our study, subjects cycled 15–20 min at 1.5 W·kg^−1^ and this might have been a too low intensity for complete BLC recovery. Adjusting the recovery intensity to 80%–100% of LT1 could have accelerated BLC recovery and improved test-retest reliability of BLC_pre_ data.

Secondly, our sample size was small (*n* = 11) compared to the recommended sample size for this type of research (*n* = ∼30) (57). Unfortunately, testing larger samples was not possible at the time of data collection due to COVID restrictions for laboratory exercise testing. Therefore, we consider our study rather a pilot study and generalization of our results should be done with care. Thirdly, we did not include a gold standard reference method for buffering capacity in our protocol to test validity of the calculated buffer capacity. The evaluation of the validity of our methods requires additional scientific investigation.

### 4.4 Conclusions

Post-exercise BLC and pH, but not pre-exercise BLC and pH, displayed good levels of reliability and can be utilized to calculate buffer capacity using maximal exercise tests of 3 to 12 min duration.

Whether the disposed methodology to assess buffer capacity will yield similar results as golden standard procedures needs further scientific investigation.

## Data Availability

The raw data supporting the conclusions of this article will be made available by the authors, without undue reservation.
