# Supplementary material for: Reliability of a non-invasive method to calculate buffer capacity after exhaustive cycling exercise of 20 s to 12 min: a pilot study
Source: Front Sports Act Living. 2025 Mar 12;7:1546117. doi: 10.3389/fspor.2025.1546117 (PMC11936892; doi:10.3389/fspor.2025.1546117)
Supplement: Supplementary file 1 [file Supplementaryfile1.docx]

Supplementary Material

# Supplementary Figures

**Supplementary Figure 1.** Pearson correlation matrix for buffer capacity $\beta1$ (**A**) and $\beta$2 (**B**). SPR1 = first isokinetic sprint; SPR2 = second isokinetic sprint; Ramp = maximal ramp test protocol; TT_3_ = 3 min maximal time trial; TT_6_ = 6 min maximal time trial; TT_12_ = 12 min maximal time trial (details see methods section). * p<0.05.

**A B**


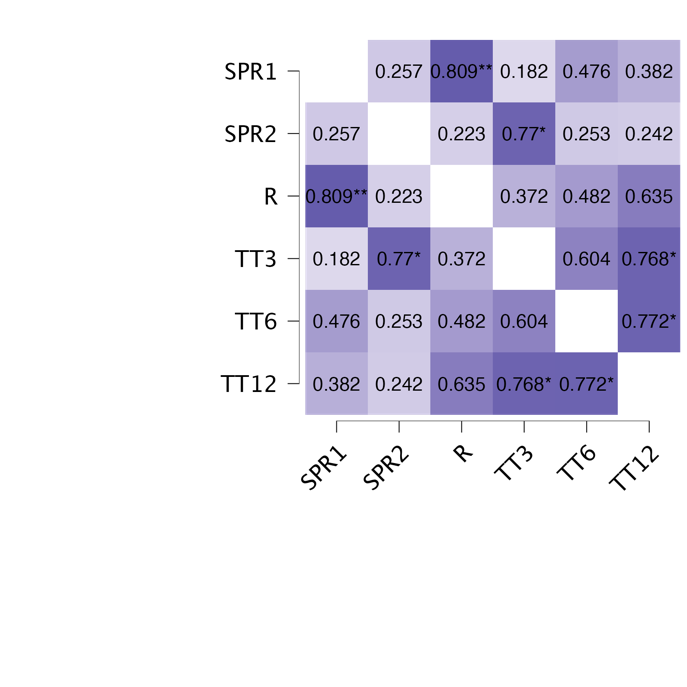

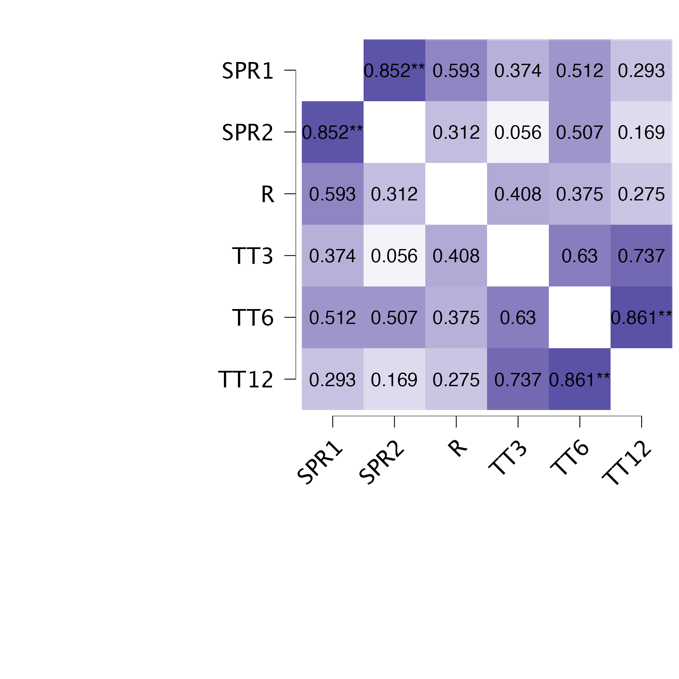


# Supplementary Tables

**Supplementary Table 1.** Average power output (W), pre (BLC_pre_) and post exercise blood lactate concentration (BLC_post_) and pH (pH_pre_, pH_post_) for the maximal performance tests (mean±SD). SPR1 = first isokinetic sprint; SPR2 = second isokinetic sprint; Ramp = maximal ramp test protocol; TT_3_ = 3 min maximal time trial; TT_6_ = 6 min maximal time trial; TT_12_ = 12 min maximal time trial (details see methods section).

|  | Power output (W) | BLC_pre_ (mmol·l^-1^) | BLC_post_ (mmol·l^-1^) | pH_pre_ | pH_post_ |
| --- | --- | --- | --- | --- | --- |
| SPR1 | 709±86 | 0.9±0.2 | 8.7±1.5 | 7.409±0.016 | 7.287±0.026 |
| SPR2 | 712±86 | 1.5±0.6 | 8.3±1.6 | 7.426±0.026 | 7.292±0.03 |
| Ramp | - | 1.3±0.5 | 11.3±2.4 | 7.419±0.017 | 7.212±0.065 |
| TT_3_ | 345±45 | 1.4±0.7 | 11.3±2.6 | 7.436±0.030 | 7.234±0.054 |
| TT_6_ | 316±44 | 1.5±0.5 | 11.8±3.0 | 7.42±0.017 | 7.185±0.088 |
| TT_12_ | 283±44 | 1.5±0.4 | 9.0±3.0 | 7.417±0.019 | 7.244±0.076 |

# Henderson-Hasselbalch equation for calculating buffer capacity

Extracellular buffer capacity can be calculated from equation (1) using the following data:

- Pre-test blood lactate concentration (BLC_pre_)
- Post- test blood lactate concentration (BLC_post_)
- Pre-test pH (pH_pre_)
- Post-test pH (pH_post_)

$\beta= \frac{\Delta\left[ HA \right]}{\Delta pH}$ (1)

$\beta= \frac{\left[ HA \right]_{post}-\left[ HA \right]_{pre}}{{pH}_{post}-{pH}_{pre}}$ (2)

Henderson-Hasselbalch equation (3):

$pH=pKa+log\left( \frac{\left[ A^{-} \right]}{\left[ HA \right]} \right)$ (3), whereas the pKa value for lactate at body temperature equals 3.87.

To calculate $\beta$ from (2), we will rearrange equation (3) to obtain $\left[ HA \right]_{pre}$ and $\left[ HA \right]_{post}$.

$pH-pKa=log\left( \frac{\left[ A^{-} \right]}{\left[ HA \right]} \right)$ (4)

$\frac{\left[ BLC \right]}{\left[ HA \right]}= {10}^{(pH-3.87)}$ (5)

Rearranging equation (5) to obtain $\left[ HA \right]$:

$\left[ HA \right]= \frac{BLC}{{10}^{\left( pH-3.87 \right)}}$ (6)

Using equation (6), $\left[ HA \right]_{pre}$ and $\left[ HA \right]_{post}$.can be calculated.

The equation for calculating obtain $\left[ HA \right]_{pre}$ and $\left[ HA \right]_{post}$ is entered into equation (2) as follows:

$\beta= \frac{\left( \frac{{BLC}_{post}}{{10}^{\left( {pH}_{post}-3.87 \right)}} - \frac{{BLC}_{pre}}{{10}^{\left( {pH}_{pre}-3.87 \right)}} \right)}{{pH}_{post}- {pH}_{pre}}$ (7)

Equation (7) has been used in this study to calculate buffer capacity ($\beta$).
